# Telemonitoring for Patients With COVID-19: Recommendations for Design and Implementation

**DOI:** 10.2196/20953

**Published:** 2020-09-02

**Authors:** Anna V Silven, Annelieke H J Petrus, María Villalobos-Quesada, Ebru Dirikgil, Carlijn R Oerlemans, Cyril P Landstra, Hileen Boosman, Hendrikus J A van Os, Marco H Blanker, Roderick W Treskes, Tobias N Bonten, Niels H Chavannes, Douwe E Atsma, Y K Onno Teng

**Affiliations:** 1 Department of Public Health and Primary Care Leiden University Medical Center Leiden Netherlands; 2 National eHealth Living Lab Leiden University Medical Center Leiden Netherlands; 3 Department of Nephrology Leiden University Medical Center Leiden Netherlands; 4 Department of Endocrinology Leiden University Medical Center Leiden Netherlands; 5 Department of Quality and Patient Safety Leiden University Medical Center Leiden Netherlands; 6 Department of Neurology Leiden University Medical Center Leiden Netherlands; 7 Department of Clinical Epidemiology Leiden University Medical Center Leiden Netherlands; 8 Department of General Practice and Elderly Care Medicine University Medical Center Groningen Groningen Netherlands; 9 Department of Cardiology Leiden University Medical Center Leiden Netherlands

**Keywords:** telemonitoring, telemedicine, eHealth, digital health, COVID-19

## Abstract

Despite significant efforts, the COVID-19 pandemic has put enormous pressure on health care systems around the world, threatening the quality of patient care. Telemonitoring offers the opportunity to carefully monitor patients with a confirmed or suspected case of COVID-19 from home and allows for the timely identification of worsening symptoms. Additionally, it may decrease the number of hospital visits and admissions, thereby reducing the use of scarce resources, optimizing health care capacity, and minimizing the risk of viral transmission. In this paper, we present a COVID-19 telemonitoring care pathway developed at a tertiary care hospital in the Netherlands, which combined the monitoring of vital parameters with video consultations for adequate clinical assessment. Additionally, we report a series of medical, scientific, organizational, and ethical recommendations that may be used as a guide for the design and implementation of telemonitoring pathways for COVID-19 and other diseases worldwide.

## Introduction

The COVID-19 pandemic has created a difficult challenge for global public health [1]. The clinical presentations of COVID-19 are highly variable, ranging from asymptomatic patients to patients with severe symptoms. Several reports indicate that patients who initially present only mild-to-moderate symptoms can show a deterioration to severe symptoms over the course of only a few days [2]. Eventually, approximately 5% of patients need respiratory support and intensive care unit admission. Patients with chronic underlying conditions are at an increased risk of such a severe course of illness when infected with COVID-19. Early identification of hypoxia and/or a deterioration of symptoms may be lifesaving [3-5]. However, in-hospital monitoring of all patients with an increased risk of severe disease puts even more pressure on health care systems that are already overwhelmed.

Telemonitoring offers the opportunity to closely monitor symptoms and vital parameters while a patient remains at home. As such, telemonitoring may enable early identification of deterioration of symptoms and allows for appropriate treatments for each patient with COVID-19. Additionally, telemonitoring may reduce the number of hospital visits and admissions, thereby decreasing the usage of personal protective material, reducing the pressure on health care personnel, and minimizing the risk of viral transmission. Telemonitoring essentially holds the promise of optimizing care for patients with (suspected) COVID-19 infection while ensuring the sustainability of health care capacity and resources for those who need it most urgently [6-8]. However, the development and implementation of a telemonitoring care pathway in the clinical workflow is challenging. The novelty of COVID-19 and the need for rapid action pose additional difficulties. Based on our extensive experiences with telemonitoring for patients with chronic diseases, we developed and implemented a telemonitoring care pathway for patients with (suspected) COVID-19 [6].

In this paper, we present our telemonitoring care pathway as an example and provide general recommendations for the implementation of telemonitoring care programs for patients with (suspected) COVID-19. Hence, the aim of this paper is to serve as a guide for the design and implementation of telemonitoring care pathways in other health care institutions, in order to optimize care and health care capacity worldwide.

## The COVID Box: An Example of a Telemonitoring Care Pathway

### Aim and Setting

**Figure 1:**
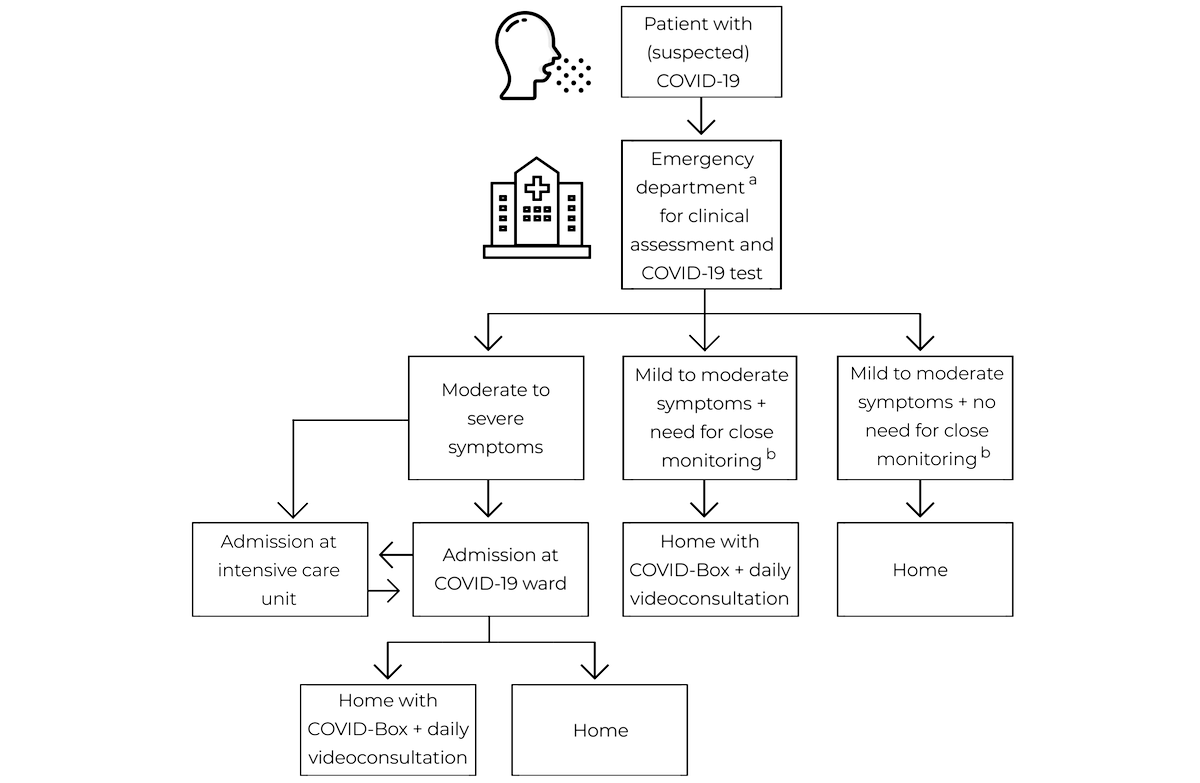
Types of patient journeys for individuals with (suspected) COVID-19. In the Netherlands, patients are only assessed at the emergency department after referral by their general practitioner or treating medical specialist (indicated by "a"). The treating physician eventually decides which patients are in need for close monitoring (indicated by "b").

**Figure 2:**
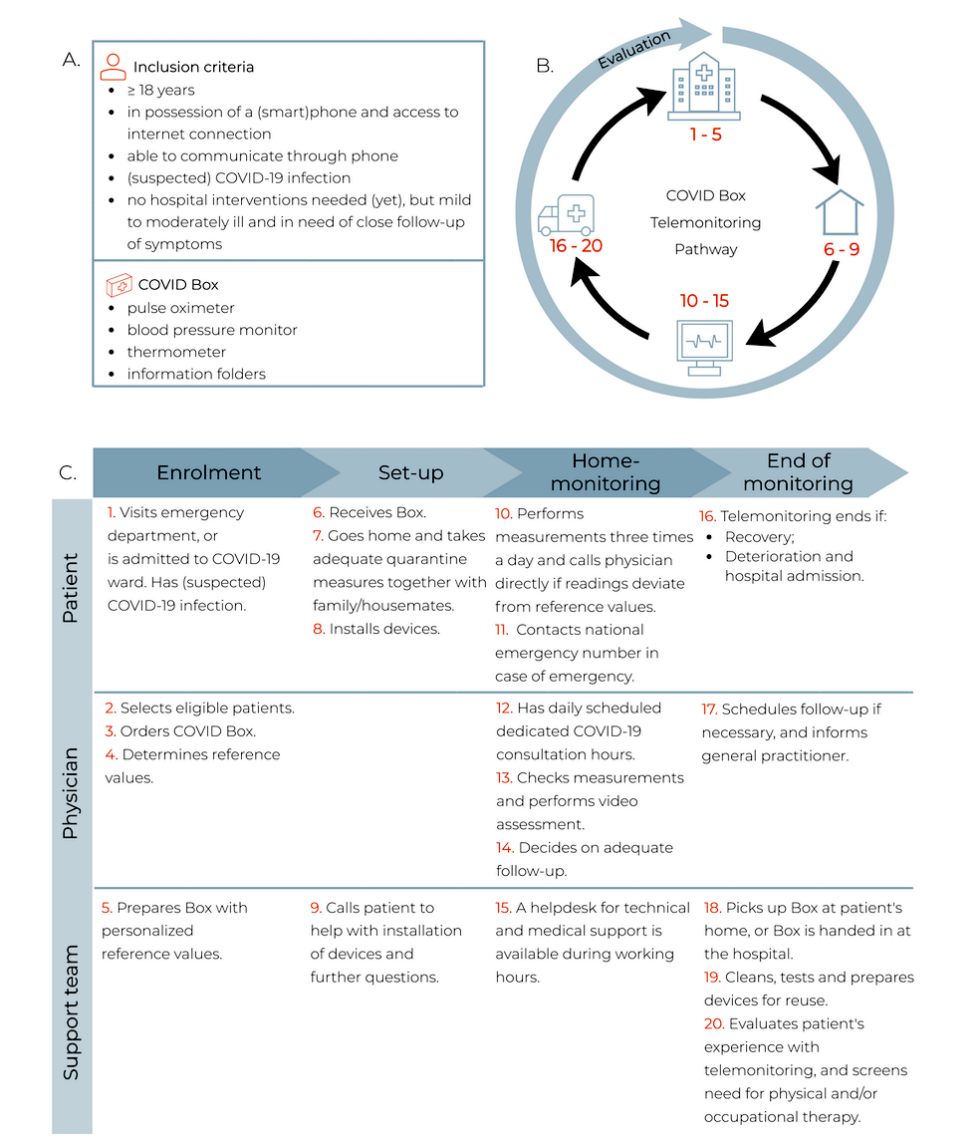
An example of a telemonitoring care pathway—the COVID Box. The COVID Box is a program developed by the Leiden University Medical Center to monitor patients with confirmed or suspected COVID-19 who have an increased risk of severe illness. (A) Inclusion criteria for the COVID Box; (B) general overview of the telemonitoring care pathway including continuous evaluation, where numbers 1-20 correspond to the steps described in (C), which provide a stepwise description of the COVID Box care pathway.

Telemonitoring for patients with (suspected) COVID-19 can be performed in many ways [9]. At our tertiary care hospital, Leiden University Medical Center (LUMC), telemonitoring is broadly applied for the management of several diseases and has been found to be a safe and effective alternative to standard care [6]. Based on this experience, the “COVID Box” telemonitoring care pathway was developed and implemented as standard care for patients with (suspected) COVID-19 over the course of a few weeks (Figures 1 and 2). The aim of this telemonitoring care pathway is to provide optimal care to patients with (suspected) COVID-19, while avoiding (unnecessary) hospitalizations and reducing the duration of hospitalizations.

### Patient Selection and Eligibility

Patients are selected for telemonitoring when visiting the emergency care department for (suspected) COVID-19 (after referral by their general practitioner), or after admission to the COVID-19 department. Patients are eligible for telemonitoring using the COVID Box in the case of a (suspected) COVID-19 infection and when exhibiting mild-to-moderate symptoms, with a need of close monitoring of vital signs and symptoms, as judged by the treating physician. In addition, psychological and social patient characteristics are taken into account.

### Home Monitoring

The COVID Box is literally a box containing a thermometer (Withings Thermo), pulse oximeter (Masimo MightySatRx), blood pressure monitor (Microlife BP B2 Basic or Withings BPM Connect), and a safety bag (for return of the devices). All devices are approved for medical use in Europe.

Patients receiving the COVID Box are instructed to measure their temperature, oxygen saturation, respiratory frequency, heart rate, and blood pressure three times a day. Patients are instructed on the use of the devices, the desired frequency of measurements, and their personalized reference values. A physician or physician assistant (supervised by a medical specialist) performs a daily video consultation to monitor the patient’s symptoms and vital parameters, whereas physical consultations are only performed when the physician or physician assistant suspects a deterioration of symptoms. Patients are instructed to contact the hospital if readings deviate from the personalized reference values or if they feel unsure or unwell. In case of an emergency, patients are advised to contact the national emergency number, and for technical support, the hospital’s telemonitoring support team should be contacted.

In the first phase of implementation, measurements of vital parameters are collected during the video consultation and entered manually into the patient’s electronic medical record (EMR). In the second phase, patients install an app, developed by the LUMC, which is linked to the devices via Bluetooth and transfers the measurements directly into the patient’s EMR.

The telemonitoring care pathway ends when patients no longer need home monitoring because symptoms have sufficiently improved, or when patients are admitted to the hospital. At such time, the devices are either picked up from the patient’s home or handed in at the hospital. Subsequently, the devices are sterilized, tested, and then reused.

### Scientific Evaluation

Between March 1 and June 15, 2020, 55 patients were monitored at home using the COVID Box. Preliminary results of the evaluation of the telemonitoring program indicate that no adverse events (ie, deaths or emergency hospital admissions) occurred among patients in the telemonitoring care pathway, and that a worsening of symptoms and need for further medical attention could be effectively detected by means of this telemonitoring care program. Eventually, 5 patients (9%) had to be admitted to the hospital due to progression of symptoms. Both patients and health care providers viewed the use of the COVID Box positively. A more detailed overview of the results of the COVID Box will be presented soon.

A prospective registry, which includes all patients who are home monitored using the COVID Box, has been designed. In this registry, baseline patient characteristics, home-monitoring data, patient-reported outcome measures, and clinical outcomes are included. Data from all patients in this registry are periodically evaluated in order to identify any adverse events in a timely manner and improve the telemonitoring care pathway.

## Recommendations for the Implementation of a COVID-19 Telemonitoring Care Pathway

The implementation of digital health initiatives has proven to be challenging [10-12]. In the last decade, several frameworks regarding the implementation of digital health have been developed [13-16]. Although these provide useful information, the onset of COVID-19 and the need for a prompt response pose additional challenges [17]. Based on our initial experiences with developing and implementing a COVID-19 telemonitoring care pathway in a tertiary care context and our extensive experience with the implementation of telemonitoring for other diseases, we provide recommendations on clinical, organizational, scientific, and ethical aspects, which can be translated into daily clinical practice (Table 1)*.* These recommendations may also be used to design and implement telemonitoring for the management of other (sub)acute diseases.

**Table 1:** Main recommendations to facilitate the implementation of a COVID-19 telemonitoring pathway.

Topic		Recommendation
**Clinical and organizational aspects**		
	Aim and setting	Define the aim of the telemonitoring program Determine whether telemonitoring will help to achieve this aim in this specific setting
	Clinical assessment	Perform initial in-person assessment (using adequate protective measures) Evaluate the appropriateness of telemonitoring for each patient, based on clinical, psychological, and social patient characteristics
	Monitoring	Determine which measurements are to be taken Generate personalized reference values and frequency of measurements for each patient Prefer video call over contact by telephone Rely on close supervision of physician rather than automated decision making Communicate to patients which actions to take in case of an emergency
	Integration in clinical workflow	Involve all stakeholders in the development and implementation process Establish solid and concise training for health care personnel Communicate availability of a telemonitoring pathway within the organization Provide support to avoid extra workload for health care personnel Organize a technical helpdesk for patients Integrate readings into the patient’s electronic medical record Evaluate the program continuously
	Resources	Reuse devices to optimize the use of resources and sustainability of the program Apply for innovation and research grants Discuss possibilities for reimbursement depending on your health financing or insurance system
**Scientific aspects**		
	Implementation	Apply scientific evidence obtained from telemonitoring chronic diseases
	Evaluation and scientific research	Perform scientific evaluation in parallel with the implementation Obtain consent to use patients' data for scientific research
**Legal and ethical aspects**		
	Privacy and data protection	Ensure intramural and extramural data security and privacy Comply with legal frameworks and clinical guidelines
	Ensuring optimal technical quality	Guarantee quality of devices and apps
	Consent and informed decisions	Ensure that the choice to use telemonitoring is a jointly made decision by both the patient and the physician; respect autonomy and patient preferences Establish responsibilities for each party involved: physician, patient, and telemonitoring team Offer alternative and opt-out options to the patient
	Equal opportunities, no discrimination	Avoid discrimination, offer equal opportunities, and plan alternative nondigital monitoring pathways

### Clinical and Organizational Considerations

#### Aim and Setting

Implementation of a new health care initiative should contribute to solving a relevant problem. In the case of COVID-19, it should optimize care for patients and relieve pressure on health care systems and professionals. In tertiary care hospitals, most patients have substantial comorbidities and vulnerabilities. As such, these patients require close monitoring and are especially suitable for telemonitoring [5]. Although telemonitoring allows for the management of patients in different settings (eg, assessing a patient’s condition and estimating the risk of COVID-19 infection in primary care), it should only be used in situations where it will add value, such as improving clinical effectiveness or satisfaction of care [9].

#### Patient Selection and Eligibility

Telemonitoring may allow for accessible and remote monitoring of a large number of patients with (suspected) COVID-19. Due to the outbreak of this new disease, our knowledge of the epidemiology, pathophysiology, and clinical course of COVID-19 is still evolving. Hence, an initial in-person assessment of the patient is recommended, especially in the case of a complex medical history.

When evaluating whether telemonitoring provides a suitable option, patients need to be assessed in a holistic manner. Psychological characteristics may influence a patient’s ability to adequately interpret measurements of vital signs and symptoms or ask for help if necessary. Social aspects, such as a patient’s home environment (eg, the possibility for home quarantine), or safety and social support (eg, for elderly patients or patients living alone) should be considered as well. Additionally, patients should feel comfortable with “digital” disease management and be capable of handling devices and interacting via video connection. Although in The Netherlands the majority of patients has access to internet and telephone, this should be asked explicitly [18,19]. Finally, patients with low (digital) health literacy require extra attention [20,21]. In contrast to telemonitoring for patients with chronic diseases, (suspected) COVID-19 patients are not accustomed to self-managing their disease and may be less likely to feel in control. Ultimately, both the physician and the patient should decide together whether remote monitoring is appropriate.

#### Home Monitoring

Given the recentness of COVID-19 and the relative inexperience with telemonitoring for managing this disease, no golden standard for how, what, and when to monitor yet exists. The use of video over telephone consultation provides extra information on general demeanor, skin color, and severity of dyspnea, and thus helps to establish a solid clinical impression [9]. The rationale to monitor respiratory rate, saturation, temperature, heart rate, and blood pressure from home is based on early warning scores developed in hospital settings [22-24]; higher scores measured outside hospital settings were also found to be associated with poorer clinical outcomes [25]. Measuring vital signs three times a day ensures recognition of gradual deterioration. Similar to in-hospital patient management, the measurement frequency and cut-off values for vital signs should be determined for each patient based on clinical judgment. If a physician decides that a patient’s parameters need to be assessed more than three times a day, this might be an indication that the patient should be admitted to the hospital to be monitored more closely. Future research should determine whether the use of continuous monitoring with wireless patches and/or automated cut-off scores could also be effective.

#### Integration With the Clinical Workflow

Irrespective of the setting, the workflow of health care professionals and supportive personnel should be adapted to a new telemonitoring care pathway. A shift from “traditional” to “digitally assisted” clinical practice is necessary. This requires experience, time, and adequate guidelines. First, solid but concise training and frequent evaluation of the process are required. Health care professionals should be informed about the availability of a telemonitoring care pathway and need dedicated time in their work schedules to follow up on their home-monitored patients. Active involvement of all stakeholders, including health care professionals and patients, in the development and implementation of telemonitoring is likely to contribute to a better support base. The extra workload associated with the development and implementation of a telemonitoring care pathway should be minimized, for example, through dedicated support teams and a helpdesk for technical problems. Finally, for patients, as well as health care professionals, it is neither efficient nor safe to log in to different systems multiple times a day. Thus, it is essential that data gathered by patients are integrated into the EMR. In addition, EMR integration facilitates the legal obligation of health care professionals to document all clinical information.

#### Resources

Telemonitoring could contribute to establishing effective, (cost-)efficient and high-quality health care. Nevertheless, it requires the availability of sufficient devices. This can be difficult, since the COVID-19 pandemic resulted in a shortage of resources. Adequate reuse of devices contributes to the optimization of resources and sustainability of the program. In our experience, patients generally hand in the devices in good working order. Additionally, permanent financing is a recurring problem for digital health solutions [15]. Reimbursement policies for telemonitoring programs vary internationally and should be further established [26]. Moreover, innovation budgets may contribute to obtaining the most appropriate devices and services. To generate scientific evidence, the availability of research grants is warranted.

### Scientific Considerations

In general, new forms of care need to be evaluated and scientifically validated before large-scale implementation. However, the current COVID-19 pandemic calls for rapid action and scientific data on telemonitoring for (sub)acute diseases and specifically for COVID-19 is scarce. Although this makes development and implementation of such a pathway difficult, we can rely on prior knowledge obtained from telemonitoring for patients with chronic diseases in order to implement telemonitoring programs that are safe and effective [6-8]. Additionally, this lack of scientific evidence underlines the urgent need for the scientific validation of these new forms of care. Scientific validation may not be top priority for health care providers at this time, but lack of validation may lead to a waste of valuable time, energy, and resources if telemonitoring does not lead to the expected benefits [27,28]. As such, scientific validation of safety and efficacy should form a key element in the development and implementation of telemonitoring, and other new forms of care, during a pandemic.

Designing a validation study for COVID-19–related telemonitoring entails several challenges. Randomized controlled trials are difficult in the early phases of a pandemic, and retrospective cohorts, which may serve as control groups, are not available. Under these conditions, assessment of the causal effect of telemonitoring on clinical endpoints is difficult. Scientific validation of the safety and (cost-)effectiveness of telemonitoring by an observational cohort study might be the best option in the early phase of the COVID-19 pandemic. This type of research can be performed in parallel to the implementation process and provides relevant insights into the clinical course of COVID-19. In the future, scientific validation can contribute to the establishment of clinical guidelines for evidence-based telemonitoring.

### Ethical Considerations

Telemonitoring involves several parties: physicians, patients, device manufacturers, and health care institutions. When considering ethical aspects, the different roles and responsibilities of these parties should be taken into account. Additionally, the COVID-19 pandemic demands a careful balance between rapid implementation and thorough ethical and legal evaluation.

#### Privacy and Data Protection

Telemonitoring brings health care to patients’ homes and therefore requires expanding the secure digital perimeter beyond health care facilities. Patients’ data need to remain accessible only for the lawful purposes of processing and exclusively to those with granted access.

The primary goal of data collection during telemonitoring is clinical. Personal health data are included in the patient’s EMR to directly provide medical treatment. Those granted access are directly involved in the treatment of the patient and are bound to professional confidentiality. Additionally, in our hospital, secondary uses of personal data generated from COVID-19 patients have been anticipated. Accordingly, assumed consent with an opt-out option has been put in place to reuse patients’ data for scientific research, a current public health priority [29]. Data obtained from patients will also be used for the evaluation of the COVID Box. From a legal perspective, activities regarding the use of sensitive information must comply with (inter)national regulations.

#### Ensuring Optimal Technical Quality

The devices and services used for telemonitoring should be user-friendly, trustworthy, validated, and approved, according to national and/or international regulations. Even if certification of quality requires time, it should not be dismissed. For example, at the time of the implementation of the COVID Box, the app had not yet been approved. In order to avoid delaying the implementation without compromising the quality of health care, the team decided to initially enter the data manually in the EMR.

#### Consent and Informed Decisions

Physicians must be aware that during a health crisis, patients may be under pressure to agree to telemonitoring. Additionally, obtaining informed consent is more complicated for COVID-19–related studies [30]. Patient and physician should engage in a dialogue about the telemonitoring system, the opt-out options, and the alternatives to telemonitoring. The choice to engage in telemonitoring requires patients to commit to a series of daily actions, and they must understand that they will assume a high level of responsibility. Therefore, it should be ensured that telemonitoring actually works in practice for the patient. Experience with current telemonitoring programs at the LUMC has shown that this type of telemonitoring is well accepted by patients and is clinically viable [6].

A telemonitoring system can be either implemented as a monitoring system in which readings are continuously monitored by the physician, or as a monitoring system in which the physician only monitors the readings once a day. The latter asks for patients to adopt an even higher level of responsibility, as they need to contact the physician themselves in case of deviating measurements. The choice for either monitoring system depends on important factors such as the patient population, the clinical features of the disease, and the local and international medical guidelines and legal frameworks.

#### Equal Opportunities and No Discrimination

Digital health should never lead to inequality or discrimination [31,32]. In the Netherlands, the possibility that patients lack the necessary technical means for telemonitoring is small [18]. However, this situation is not the same in all countries. Additionally, low literacy regarding (digital) health could be a significant barrier to telemonitoring [33,34]. The availability of an intuitive, readily accessible, and inclusive app, which automatically collects measurements, could be an effective and user-friendly solution to avoid inequality and discrimination. Furthermore, keeping an alternative “analogue” monitoring pathway readily available might contribute to offering equal opportunities to all patients.

Finally, a patient's decision to reject telemonitoring may be based on other personal reasons (eg, lack of privacy at home). Independent of the motives, which may or may not be explained to the physician, the autonomous choice of the patient should be respected.

## Conclusion

Innovative digital strategies such as telemonitoring have great potential to improve the management of COVID-19. Telemonitoring may optimize care for patients with COVID-19 by detecting clinical deterioration at an early stage. Additionally, telemonitoring reduces the number of hospital visits and admissions, thereby enabling the efficient use of scarce health care resources and lowering the risk of further transmission of the virus. The direct evidence to support the use of telemonitoring for COVID-19 is still being gathered and analyzed, but preliminary data and previous experiences with other diseases indicate that telemonitoring can be an important tool during the pandemic.

This paper presents a specific telemonitoring care pathway for COVID-19 and offers a set of medical, scientific, organizational, and ethical recommendations. These recommendations may be used as a guide for the design and implementation of telemonitoring for patients with a confirmed or suspected case of COVID-19, and may also be used for the design and implementation of telemonitoring care pathways for other diseases.

## Abbreviations

EMR: electronic medical record
LUMC: Leiden University Medical Center
